# Tumor-Specific Pro-Thrombotic Gene Expression in Head and Neck Squamous Cell Carcinoma: A Multi-Cohort Transcriptomic Analysis

**DOI:** 10.3390/cancers18071055

**Published:** 2026-03-25

**Authors:** Kiranya E. Arnold, Nadia Debick, John Brognard, Auyon J. Ghosh

**Affiliations:** 1 Department of Otolaryngology, SUNY Upstate Medical University, Syracuse, NY 13210, USA; debickn@upstate.edu; 2 Department of Surgery, SUNY Upstate Medical University, Syracuse, NY 13210, USA; brognarj@upstate.edu; 3 Department of Medicine, SUNY Upstate Medical University, Syracuse, NY 13210, USA

**Keywords:** venous thromboembolism, head and neck cancer, coagulome, tumor microenvironment, gene expression

## Abstract

**Objectives**: Head and neck tumors have been associated with varying risks for venous thromboembolism (VTE). Through a cross-tumor comparison, we assessed site-specific coagulation-related gene expression changes in head and neck squamous cell carcinoma (HNSCC) compared to squamous cell tumors in the esophagus (ESCCa) and lung (LUSC). Further, we assessed the relationship between clinicopathologic features of HNSCC and coagulome gene expression. **Methods**: RNA-sequencing data from primary tumor tissues of HNSCCa, ESCCa, and LUSC were obtained from The Cancer Genome Atlas (TCGA). Three previously identified pro-thrombotic genes (*F3*, *SERPINE1*, and *SERPINB2*) were analyzed and, for pan-cancer comparisons, gene expression was Z-standardized and summarized as a composite coagulome score. For HNSCCa-specific analyses, gene expression was compared using log2 RSEM counts, contrasting between HPV status, primary tumor site, tumor stage, grade, and demographic characteristics. **Results**: HNSCCa demonstrated the highest composite coagulome activation (mean Z-score = 0.29, 95% CI: 0.23–0.35) compared with LUSC and ESCCa (mean Z-scores = −0.27 and −0.16, respectively; *p* < 0.001). Among 487 HNSCCa tumors, HPV-negative tumors exhibited significantly higher composite coagulome expression than HPV-positive tumors (mean ± SD, 11.25 ± 1.39 vs. 10.14 ± 1.30; *p* < 0.001). Oral cavity tumors demonstrated the highest coagulome expression, while oropharyngeal tumors were most suppressed. Higher histologic grade was inversely associated with coagulome expression (*p* < 0.001). Patient age, sex, and race were not significantly associated with coagulome expression. **Conclusions**: HNSCCa exhibits a tumor-specific pro-thrombotic expression profile with substantial heterogeneity driven by HPV status and primary tumor site. Despite elevated tumor-specific pro-coagulant signaling, these findings reflect tumor-specific pro-thrombotic potential rather than clinical VTE risk in HNSCCa, which likely remains context-dependent and may require additional inflammatory or treatment-related triggers to clinically manifest.

## Simple Summary

The risk for venous thromboembolism (VTE) in patients with head and neck squamous cell carcinoma (HNSCCa) is not fully defined. In this study, we aimed to better understand the risk of VTE by comparing coagulation-related gene expression levels between squamous cell carcinomas of the head and neck, esophagus, and lung. We also compared gene expression levels among different subtypes of head and neck cancer. This research may help inform future strategies for personalized postoperative VTE prevention in patients with HNSCCa.

## 1. Introduction

Different solid tumors are associated with varying risks of venous thromboembolism (VTE), with glioblastoma multiforme (GBM) and pancreatic adenocarcinoma (PAAD) considered to have the highest risk of VTE [1,2]. Unlike GBM and PAAD, tumors of the head and neck are not traditionally classified as high-risk for VTE, yet reported VTE incidences reach up to 33% in individuals with head and neck cancer [3,4,5,6,7]. Head and neck cancers comprise a diverse group of malignancies, with squamous cell carcinoma (HNSCCa) accounting for more than 90% of tumors [8]. Despite similar histologic features, there appears to be substantial biological and anatomic heterogeneity in VTE risk between primary sites within the head and neck region [7,9]. Whether these observed differences in VTE risk are partially due to variations in the prothrombotic properties of the primary tumor tissue has not been explored.

The tumor coagulome concept refers to the balance between procoagulant activity and fibrinolysis within the tumor microenvironment (TME) and describes the key effectors—genes and proteins—contributing to this balance [10,11,12,13,14]. Cancer cells directly provoke hypercoagulability of the TME by releasing tissue factor (TF), a major procoagulant encoded by *F3* [15,16]. Prior work suggests that oral cavity squamous cell carcinoma (OSCCa) demonstrates both high procoagulant (*F3*) and fibrinolytic activity [14,17]. In certain tumor types, circulating *F3* is positively correlated with increased rates of cancer-associated thrombosis (CAT) [14,15,18]. For example, VTE rates in PAAD have been shown to be higher in tumors with high TF expression compared with those with low TF expression [19]. However, the association of TF with increased clinical VTE risk is highly variable and many tumor types do not demonstrate a correlation between TF expression and increased VTE [20,21]. Whether increased expression of TF is linked to higher rates of VTE among patients with HNSSCCa remains unknown. While prior work has described tumor coagulome patterns across multiple cancers and within OSCCa specifically, it remains unclear whether these patterns extend across the broader spectrum of HNSCCa or how they compare with other squamous cell carcinomas arising from anatomically distinct sites. In addition, the relationship between coagulome activation and key clinical determinants of HNSCCa biology—including human papilloma virus (HPV) status, primary tumor site, and pathologic stage—has not been systematically evaluated within HNSCCa.

In this study, we used RNA sequencing (RNA-seq) data from primary tumor tissues in the Cancer Genome Atlas (TCGA) to compare mRNA expression of three key pro-coagulant genes (*F3*, *SERPINE1*, and *SERPINB2*), which were adopted from a previously described pro-coagulant gene set [14,17]. *SERPINE1* and *SERPINB2* are anti-fibrinolytic genes encoding plasminogen activator inhibitor-1 (PAI-1) and plasminogen activator inhibitor-2 (PAI-2), respectively. The objective was to perform a cross-tumor comparison and test whether previously identified site-specific coagulome patterns identified in OSCCa extend across all HNSCCa, and whether these patterns differ from other solid tumors with similar histology and risk factors, specifically esophageal squamous cell carcinoma (ESCCa) and lung squamous cell carcinoma (LUSC) [22,23,24]. We additionally sought to evaluate associations between clinical and pathologic features of HNSCCa as they relate to coagulome gene expression, using standardized composite coagulome scores and HPV-stratified analyses to provide a more detailed characterization of tumor-specific pro-thrombotic biology across squamous cell carcinomas.

## 2. Materials and Methods

### 2.1. Data Sources and RNA-Seq Data Acquisition

This study was exempt by the State University of New York Upstate Medical University institutional review board. RNA-seq expression data and associated clinical annotations for HNSCCa, ESCCa, and LUSC were accessed from TCGA via the UCSC Xena data portal (https://xenabrowser.net) on 20 November 2025. Study details for TCGA have previously been published; briefly, TCGA enrolled patients with newly diagnosed, treatment-naïve malignancies who provided informed consent for comprehensive molecular profiling of tumor specimens [25,26]. Tumor samples were collected at participating institutions, centrally processed, and subjected to standardized quality control, sequencing, and clinical annotation procedures prior to public release. TCGA sample barcodes were used to identify and retain primary tumor samples (sample type code “01”). Samples corresponding to normal adjacent tissue (“11”) or metastatic tissue (“06”) were excluded to ensure evaluation of tumor-specific gene expression. Tumor type was coded as HNSCCa, LUSC, or ESCCa. Only primary tumor samples with complete available expression data were included.

For HNSCCa, additional clinical and pathologic data (HPV status, T-stage, N-stage, histologic grade, age, sex, race, and ICD-10 site) were obtained from cBioportal (https://www.cbioportal.org) via the TCGA-HNSC PanCancer Atlas dataset [27,28]. HPV status was defined based on either *p16* immunohistochemistry or HPV DNA/RNA testing, as reported by TCGA.

### 2.2. Pro-Thrombotic Coagulome Gene Selection and Expression Quantification

Three pro-thrombotic genes previously associated with tumor-mediated coagulation of solid tumors were selected based on previous literature as described by Saidak et al. and Lottin et al. [14,17]. These included *F3* (tissue factor), *SERPINE1* (PAI-1), and *SERPINB2* (PAI-2). These genes were selected from a previously described six-gene tumor coagulome panel (*F3*, *PLAT*, *PLAU*, *PLAUR*, *SERPINE1*, and *SERPINB2*), with the present analysis focusing specifically on the pro-thrombotic components of this panel to characterize tumor-associated pro-coagulant signaling and thrombin generation potential [14,17].

Normalized bulk RNA-sequencing expression counts for *F3*, *SERPINE1*, and *SERPINB2* were selected from TCGA counts matrices in log2-transformed RNA-Seq by Expectation-Maximization (RSEM) format, which provides gene expression counts normalized for sequencing depth and transcript length, as provided by UCSC Xena platform. For pan-cancer analyses, counts were standardized using gene-wise Z-scores, computed by subtracting the global mean and dividing by the global standard deviation, yielding gene-specific Z-scores. A composite coagulome score was then calculated for each tumor sample by averaging the three gene-specific Z-scores (*F3*, *SERPINE1*, and *SERPINB2*), enabling direct comparison of global coagulome activation between tumor types. Composite coagulome scores and individual gene Z-scores were summarized separately for HNSCCa, ESCCa, and LUSC.

For HNSCCa-specific analyses, log2-transformed RSEM counts without Z-standardization were used. A composite coagulome score for HNSCCa analyses was calculated as the mean log2 RSEM expression of *F3*, *SERPINE1*, and *SERPINB2* within each tumor sample.

### 2.3. Clinical Variables and Tumor Site Classification

HNSCCa clinicopathologic variables were restricted to TCGA HNSCCa tumors with complete clinical annotation from cBioPortal and included HPV status (positive/negative), ICD-10 primary site (C00–C14, C32), T stage and N stage (American Joint Committee on Cancer [AJCC], 8th edition), histologic grade (G1–G2 vs. G3), age (continuous), sex, and race. For ICD-10 analyses, subcodes were collapsed to three-character categories (e.g., C02) and grouped according to anatomic regions defined by the AJCC as follows: oral cavity (C00, C02–C06); oropharynx (C01, C09, C10); larynx (C32); and other/overlapping sites and hypopharynx (C13, C14). Hypopharyngeal and overlapping pharyngeal sites were grouped due to small sample sizes within TCGA, which precluded reliable site-specific analyses. Analyses excluded rows with missing values for the variables required in a given comparison.

### 2.4. Statistical Analysis

Differences in composite coagulome scores (composite Z-scores for pan-cancer analyses) and individual gene-level Z-scores between tumor types were evaluated using the Kruskal–Wallis H test. When global tests were significant, pairwise comparisons between tumor types (HNSCCa vs. LUSC, HNSCCa vs. ESCCa, and LUSC vs. ESCCa) were performed using Mann–Whitney U tests with Bonferroni correction for multiple comparisons, with an adjusted significance threshold of *p* < 0.0167. For pan-cancer analyses, results are summarized as mean Z-scores with 95% confidence intervals (95% CIs) and corresponding global and pairwise Bonferroni-adjusted *p*-values to describe group-level differences in standardized expression.

For HNSCCa-specific analyses, group comparisons (e.g., HPV, sex, grade, and tumor stage) and association with individual gene expression (*F3*, *SERPINE1*, *SERPINB2*) and composite coagulome score were performed using two-sided Mann–Whitney U or Kruskal–Wallis H tests as appropriate. Spearman rank correlation was used for continuous variables (e.g., age). To account for multiple comparisons in the HNSCCa-specific analyses, Benjamini–Hochberg false discovery rate (FDR) correction was applied across all HNSCCa-specific inferential tests, and adjusted *p*-values are reported in Supplementary Table S2. Accordingly, HNSCCa-specific results are reported using non-parametric test statistics (U, H, or ρ) and *p*-values, with expression values summarized as mean ± standard deviation (SD) with 95% CI to facilitate interpretation given the large sample size and approximately symmetric distributions.

Statistical significance was defined as two-sided *p* < 0.05, with adjustment for multiple comparisons applied where appropriate. All analyses were conducted using Python (version 3.11; pandas, NumPy, SciPy) in November 2025.

## 3. Results

### 3.1. Comparative Coagulome Activation Across Squamous Cell Carcinomas

A total of 1117 tumor samples from three squamous cell carcinoma primary sites were analyzed: HNSCC (*n* = 520), ESCC (*n* = 95), and LUSC (*n* = 502). Coagulome activation differed significantly across tumor types (Kruskal–Wallis, *p* < 0.001) (Table 1). Bonferroni-adjusted pairwise comparisons demonstrated significantly higher coagulome activation in HNSCCa compared with both LUSC (*p* < 0.001) and ESCCa (*p* < 0.001). No significant difference was observed between LUSC and ESCCa (*p* = 0.35).

**Figure 1:**
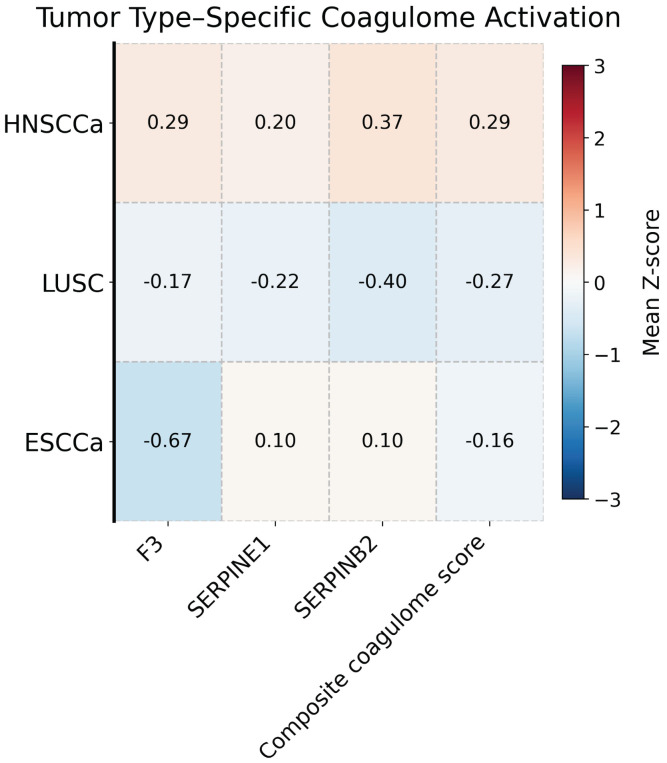
Heat map comparison of pro-coagulant gene expression across three squamous cell carcinomas (HNSCCa, ESCCa, LUSC).

Coagulome activation differed distinctly across tumor types (Figure 1). HNSCCa tumors demonstrated the highest composite coagulome activation (mean Z-score = 0.29, 95% CI: 0.23–0.35), while LUSC and ESCCa exhibited suppressed coagulome signatures (mean Z-scores = −0.27 and −0.16, respectively). Individual gene-level analyses showed the same directional pattern: HNSCCa had the highest expression of *F3*, *SERPINE1*, and *SERPINB2* compared with other squamous cell carcinoma sites (all global KW *p* < 0.001; Table 1). These findings indicate that squamous carcinomas arising from different anatomic sites display distinct pro-thrombotic molecular phenotypes, with HNSCCa showing the strongest activation of the procoagulant coagulome.

### 3.2. HPV Status Strongly Influences Coagulome Expression in HNSCCa

**Figure 2:**
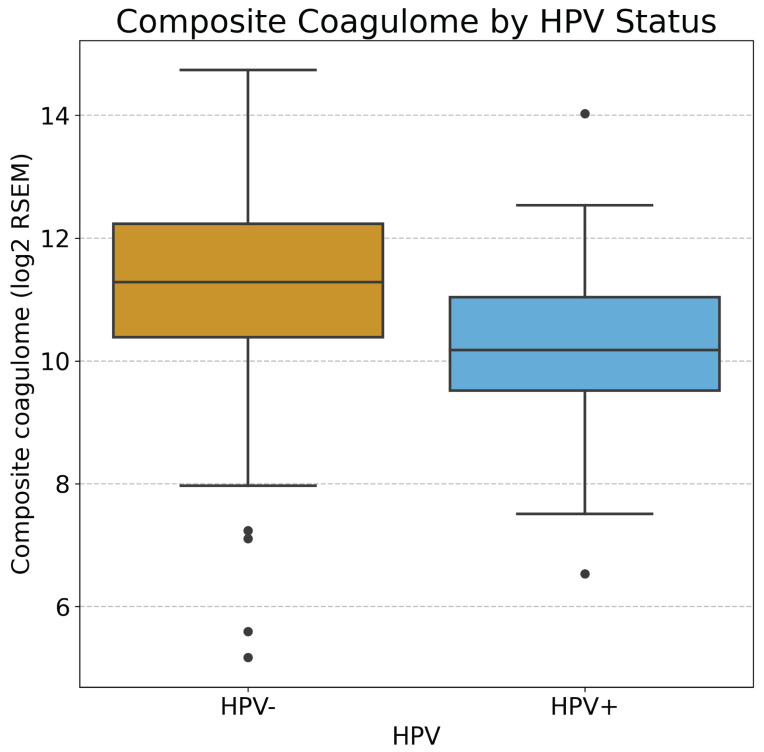
Boxplot showing expression of composite coagulome according to HPV status in HNSCCa.

Among 487 evaluable tumors (HPV+ *n* = 72; HPV − *n* = 415), HPV-negative tumors showed significantly higher coagulome activation using raw expression values (Table 2). The composite coagulome score was significantly higher in HPV− versus HPV + tumors (mean ± SD: 11.25 ± 1.39 vs. 10.14 ± 1.30; *p* < 0.001) (Figure 2). Compared to HPV+ tumors, HPV− subtypes demonstrated significantly higher expression of *F3* (mean: 11.47 ± 1.71 vs. 10.23 ± 1.79; *p* < 0.001) and *SERPINE1* (mean: 12.65 ± 1.60 vs. 10.38 ± 1.53; *p* < 0.001). *SERPINB2* did not differ significantly by HPV status (mean: 9.63 ± 2.51 vs. 9.82 ± 2.00; *p* = 0.83).

### 3.3. Primary Tumor Site Demonstrates Distinct Coagulome Signatures in HNSCCa

**Figure 3:**
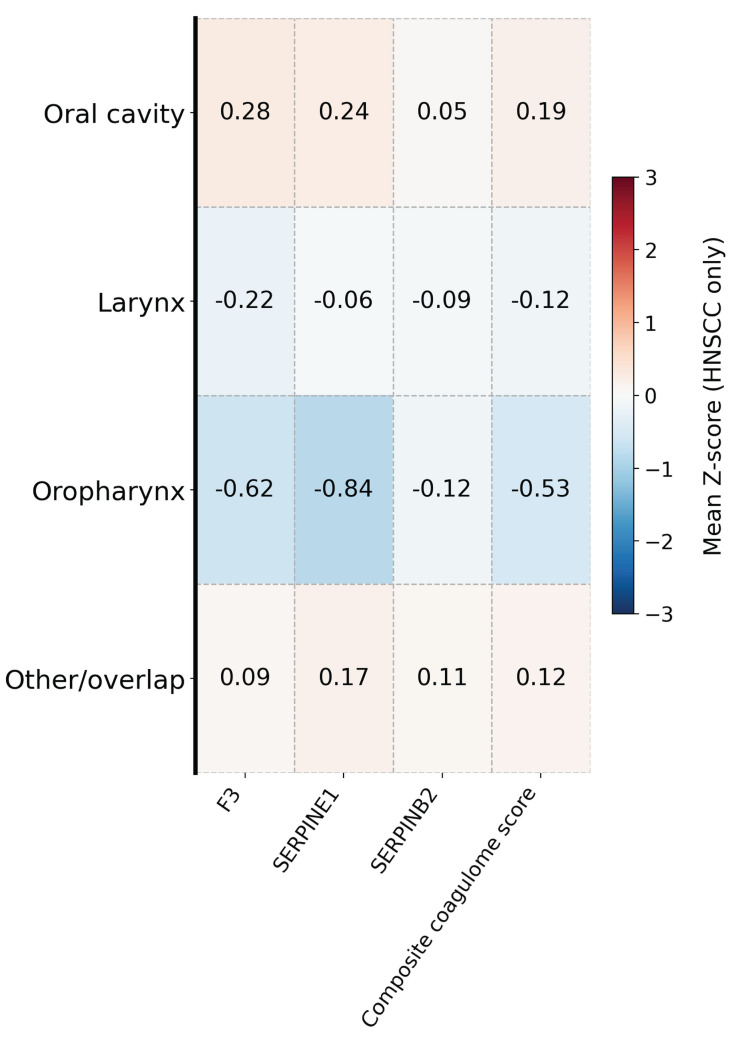
Heat map of HNSCCa primary tumor site-specific differences in pro-coagulant gene expression and composite coagulome score, with expression values shown as Z-standardized scores for visualization.

Analysis by tumor subsite revealed significant variation in composite coagulome activation across primary sites (Kruskal–Wallis *p* < 0.001) (Table 3, Figure 3). Among individual genes, expression of *F3* and *SERPINE1* differed significantly across primary sites, with the highest mean expression observed among oral cavity tumors (Kruskal–Wallis *p* < 0.001 for all) (Table 4). Oropharyngeal tumors consistently showed the most suppressed coagulome signature across these markers. *SERPINB2* expression did not significantly differ across primary sites (*p* = 0.17), although mean levels were numerically highest within the other/overlap/hypopharynx category.

Among HPV-negative tumors, primary site remained significantly associated with pro-thrombotic gene expression (all global Kruskal–Wallis *p* < 0.05) (Supplementary Table S1). Oral cavity tumors demonstrated the highest overall coagulome activation (mean ± SD: 11.46 ± 1.35), driven by elevated expression of *F3* (mean ± SD: 11.78 ± 1.57) and *SERPINE1* (mean ± SD: 12.81 ± 1.55). Laryngeal tumors showed modestly suppressed expression across all markers. Oropharyngeal tumors exhibited a heterogeneous profile, characterized by lower *F3* expression (mean ± SD: 10.99 ± 1.52) and markedly reduced *SERPINB2* (mean ± SD: 8.48 ± 2.71), despite relatively higher *SERPINE1* expression (mean ±SD: 12.97 ± 1.59). Tumors grouped as other/overlap/hypopharynx showed intermediate activation (mean ± SD: 11.39 ± 1.51) with consistently elevated *SERPINE1* (mean ± SD: 12.71 ± 1.49).

### 3.4. Correlation Between HNSCCa Coagulome Expression and Pathologic Stage and Histologic Grade

When stratified by T stage, advanced tumors (T3–T4) exhibited increased expression of *SERPINE1* compared with early-stage tumors (mean ± SD: 12.59 ± 1.57 vs. 12.11 ± 1.84; *p* = 0.0056) (Table 5). The composite coagulome score showed a modest, nonsignificant shift from 11.04 ± 1.34 in T1–T2 tumors to 11.16 ± 1.42 in T3–T4 tumors (*p* = 0.27). No significant differences were observed for *F3* or *SERPINB2* across T stage.

Across nodal stages, composite coagulome expression was significantly higher in N0 tumors compared with N1–N3 tumors (mean ± SD: 11.29 ± 1.35 vs. 11.00 ± 1.45; *p* = 0.014). The largest nodal-stage effect was observed for *SERPINB2*, which fell from 10.09 ± 2.34 in N0 tumors to 9.43 ± 2.44 in N1–N3 tumors (*p* = 0.0010). No other gene exhibited a significant association with nodal burden.

Poorly differentiated tumors (Grade 3) exhibited significantly lower coagulome activation compared with well or moderately differentiated tumors (Grade 1–2). Mean composite coagulome score declined from 11.29 ± 1.35 in G1–2 tumors to 10.56 ± 1.45 in G3 tumors (*p* < 0.001). This pattern was most strongly driven by reductions in *SERPINB2* (9.99 ± 2.28 vs. 8.75 ± 2.52; *p* < 0.001) and *F3* (11.40 ± 1.65 vs. 10.94 ± 2.02; *p* = 0.037). *SERPINE1* showed a similar downward trend (12.47 ± 1.56 vs. 11.98 ± 2.11) but did not achieve statistical significance (*p* = 0.070).

### 3.5. Clinical and Demographic Characteristics Show Minimal Association with HNSCCa Coagulome Expression

No correlation was observed between patient age and composite coagulome activation or individual gene expression (Supplementary Table S2). Coagulome expression was comparable between male and female individuals (Mann–Whitney U test, *p* = 0.28). Expression of *F3*, *SERPINE1*, and *SERPINB2* also did not differ by sex (all *p* > 0.30). Race showed no significant association with composite coagulome expression or individual coagulome genes (Kruskal–Wallis tests, all *p* > 0.10).

### 3.6. FDR-Adjusted HNSCCa-Specific Analyses

After Benjamini–Hochberg FDR correction, most associations remained significant, with the exception of the modest association between tumor grade and *F3* expression (Supplementary Table S3).

## 4. Discussion

Among squamous cell carcinoma from the three anatomically distinct sites analyzed, HNSCCa demonstrated the highest overall pro-thrombotic gene signature, with elevated expression of *F3*, *SERPINE1*, and *SERPINB2* compared with ESCCa and LUSC. These findings suggest that head and neck tumors have intrinsic molecular features consistent with increased thrombin generation (*F3*) and impaired fibrinolysis (*SERPINE1* and *SERPINB2*), reflecting pro-thrombotic potential rather than absolute VTE risk. Because TCGA lacks clinical outcome data, these observations should be interpreted as hypothesis-generating rather than evidence of increased clinical thrombosis risk. Although ESSCa and LUSC have a higher clinical incidence of VTE compared to HNSCCa, their comparatively reduced tumor-specific pro-thrombotic signature suggests that thrombosis in these cancers could be driven more by patient-specific clinical factors (e.g., systemic treatment, malnutrition, and indwelling catheters) rather than tumor-specific biology.

We found HPV status to be the single strongest determinant of pro-thrombotic signaling among HNSCCa, with HPV-negative tumors demonstrating higher overall coagulome activation and markedly higher expression of TF (*F3*) and PAI-1 (*SERPINE1*) compared with HPV-positive tumors. These findings are consistent with previous studies, which found lower expression of *F3* among HPV-positive tumors, and support the idea that viral oncoprotein-driven tumors exhibit fundamentally different inflammatory, immune, and pro-coagulant biology compared with HPV-independent tumors [29]. The absence of a statistically significant difference in *SERPINB2* expression by HPV status may reflect differences in cellular composition within the TME. Whereas *SERPINE1* reflects tumor-intrinsic stromal and inflammatory pathways—features characteristic of HPV-negative tumors—*SERPINB2* is more closely associated with immune cell populations, such as those characteristic of HPV-positive tumors [30,31,32,33]. As a result, increased immune cell-derived *SERPINB2* in HPV-positive tumors may offset tumor-intrinsic differences, resulting in a net neutral effect in bulk RNA analyses. Additionally, differences in immune infiltration and tumor purity between HPV-positive and HPV-negative tumors may influence bulk expression estimates and should be considered when interpreting these findings.

Among primary head and neck tumor sites, we observed substantial heterogeneity in tumor-specific pro-thrombotic signaling. Tumors originating from the oral cavity and other/overlapping sites (including hypopharynx) exhibited the highest pro-thrombotic gene expression with significantly elevated levels of *F3* and *SERPINE1* compared to tumors of the larynx and oropharynx, which demonstrated an overall reduced coagulome profile. A sensitivity analysis limited to HPV-negative tumors confirmed site-specific differences and further revealed elevated expression of *F3* and *SERPINE1* among OSCCa tumors, with more modest *SERPINB2* elevation, and a similar but attenuated pattern in other/overlapping sites (including hypopharynx). While previous reports have shown a higher level of *F3* expression among OSCCa [17], they did not report on anti-fibrinolytic gene expression (*SERPINE1* and *SERPINB2*). Our analysis further expands upon these findings by demonstrating a distinct pro-coagulant signature among HPV-negative OSCCa, characterized by coordinated upregulation of pro-thrombotic (*F3*) and anti-fibrinolytic (*SERPINE1* and *SERPINB2*) genes. These findings reinforce the biologic distinction of OSCCa as a tumor subtype with an intrinsically pro-thrombotic microenvironment [29].

Interestingly, we found that coagulome regulation did not appear to be governed by a single dominant gene, but rather reflected context-dependent variation across individual components. For example, higher tumor grade was inversely associated with overall composite coagulome activation, driven primarily by reductions in *SERPINB2* and *F3*. The biological mechanisms for this observation remain unclear; however, we suspect it may reflect reduced stromal content and greater de-differentiation among higher-grade tumors, which could lead to diminished expression of stromal and epithelial pro-coagulant genes. In addition, our results indicate modest and inconsistent effects of T-stage and nodal status on pro-thrombotic gene expression (*SERPINE1* increased with T-stage; *SERPINB2* decreased with nodal involvement). This contrasts with prior reports of the tumor coagulome in OSCCa, which found no association between pro-thrombotic gene expression and histologic grade, or T- or N-stage [17]. While this discrepancy may be explained by the limited pro-coagulant profile evaluated by Lottin et al. (*F3* expression only), our findings suggest that intrinsic tumor biology—rather than histologic grade or locoregional extent of disease—is the principal driver of overall coagulome regulation in HNSCCa, with advanced T-stage contributing to a lesser degree [17]. This is particularly important in OSCCa, where complete tumor resection is the primary recommended treatment for all surgically resectable tumors and is frequently associated with long operative times, complex reconstruction, and prolonged recovery. These factors, combined with the observed pro-thrombotic phenotype, raise the possibility that tumor-specific biology may contribute to perioperative thrombotic risk in a subset of patients with HNSCCa. However, given the absence of correlative clinical data and ongoing concerns regarding bleeding risk in major head and neck surgery, these findings should be interpreted as hypothesis-generating but may nonetheless provide a biologic rationale for prospective studies evaluating risk-adapted thromboprophylaxis strategies.

Although our findings may appear discordant with clinical VTE rates observed across these tumor types, they may help explain the heterogeneity of VTE risk among head and neck cancer patients [8]. For example, patients with thyroid or salivary gland cancer are thought to have reduced risk of VTE compared to other cancers of the upper aerodigestive tract [7]. Prior work has postulated that this inconsistency may be due to high levels of both pro-thrombotic and pro-fibrinolytic genes, resulting in a net neutral risk of thrombosis [14,17]. In addition, we did not observe any significant associations between pro-thrombotic gene expression and clinical or demographic characteristics such as age, sex, or race in HNSCCa tumors [34,35]. Taken together, these findings suggest that additional pro-thrombotic or anti-fibrinolytic pathways beyond *F3*, *SERPINE1*, and *SERPINB2* may contribute to clinical VTE risk, or that tumor-specific signaling represents only one component of a multifactorial thrombotic process in HNSCCa. Moreover, because this restricted gene signature focuses on tumor-intrinsic pro-coagulant signaling, it may not capture other relevant thrombotic mechanisms such as platelet activation, complement signaling, neutrophil extracellular traps, or endothelial activation.

### Limitations

This study has several limitations. First, TCGA tumor specimens represent treatment-naïve primary tumors collected at the time of initial biopsy or resection. As a result, the transcriptional profiles analyzed here do not capture the substantial pro-thrombotic changes that occur with real-world treatment—such as chemoradiation, prolonged hospitalization, infection, immobility, or postoperative inflammation—which are important drivers of VTE risk. In addition, bulk RNA-sequencing reflects baseline transcript abundance rather than functional activity; it does not measure cell-surface tissue factor activity, PAI-1/PAI-2 inhibitory capacity, stromal–immune interactions, or platelet- and endothelial-derived contributors to thrombosis. Our pro-thrombotic “coagulome” markers were limited to *F3*, *SERPINE1*, and *SERPINB2*, and therefore only capture a subset of the broader coagulation and fibrinolytic pathways involved in tumor-associated thrombosis.

Interpretation is further constrained by several analytic factors. HPV status in TCGA is assigned through variable methods with imperfect concordance, creating potential misclassification bias. Primary site designation is based on ICD-10 coding, which often groups tumors into “other” or “overlapping” categories; samples from hypopharynx and less common sites were combined due to small numbers, which may obscure site-specific biology. Bulk RNA-seq cannot distinguish tumor from stromal or immune cell expression, and the age of the dataset limits generalizability to newer treatment practices such as use of immunotherapy and post-operative chemoprophylaxis among some head and neck surgeons [36]. Lastly, because TCGA lacks clinical VTE outcomes, we could not correlate gene expression with actual thrombosis events, anticoagulation use, perioperative factors, or other important contributors to VTE risk in patients with cancer.

## 5. Conclusions

HNSCCa exhibits a distinct tumor-specific, pro-thrombotic phenotype with marked clinical variation according to HPV status and primary site. Our findings show that while pro-coagulant gene expression contributes to baseline thrombotic potential, it does not fully account for the comparatively low VTE rates reported among individuals with HNSCCa. This suggests that transcriptional pro-thrombotic activity alone may be insufficient to explain clinically significant thrombosis in HNSCCa. We propose that VTE risk in HNSCCa is likely context-dependent: tumor-specific pro-coagulant signaling may remain biologically primed but only becomes clinically relevant in the presence of external inflammatory or treatment-related triggers (e.g., major surgery, postoperative cytokine surges, infection, or radiation). This model aligns with the unique VTE phenotype observed in head and neck cancer—generally low at baseline but increased in high-inflammation settings such as extensive free-flap surgery [37,38,39]. These findings highlight the need for VTE-prevention strategies in HNSCCa that consider both tumor biology and treatment context. Future work integrating tumor transcriptomics with plasma proteomics, perioperative cytokine profiling, and prospective clinical outcomes will be essential to determine whether coagulome signatures can serve as meaningful biomarkers for individualized thromboprophylaxis or early intervention.

## Figures and Tables

**Table 1:** Mean Standardized Coagulome Expression (Z-scores) Across Tumor Types.

Variable	HNSCCa Mean (95% CI)	LUSC Mean (95% CI)	ESCCa Mean (95% CI)	Sample Size (HNSCCa/ LUSC/ESCCa)	KW (H Statistic)	KW *p*-Value	Adjusted Pairwise *p*-Values
Composite Coagulome (mean Z)	0.29 (0.23–0.35)	−0.27 (−0.32–−0.21)	−0.16 (−0.29–−0.03)	520/502/95	153.01	5.94 × 10^−34^	H-L: 1.97 × 10^−33^ * H-E: 9.23 × 10^−08^ * L-E: 0.35
Z_F3	0.29 (0.20–0.38)	−0.17 (−0.25–−0.10)	−0.67 (−0.85–−0.49)	520/502/95	103.63	3.13 × 10^−23^	H-L: 1.06 × 10^−14^ * H-E: 1.38 × 10^−15^ * L-E: 2.19 × 10^−05^ *
Z_SERPINE1	0.20 (0.12–0.29)	−0.22 (−0.30–−0.15)	0.10 (−0.12–0.32)	520/502/95	56.01	6.88 × 10^−13^	H-L: 4.26 × 10^−13^ * H-E: 1.00 L-E: 3.92 × 10^−3^ *
Z_SERPINB2	0.37 (0.30–0.44)	−0.40 (−0.49–−0.31)	0.10 (−0.10–0.29)	520/502/95	147.85	7.84 × 10^−33^	H-L: 2.05 × 10^−33^ * H-E: 4.56 × 10^−2^ * L-E: 5.40 × 10^−05^ *

Abbrev. HNSCCa, head and neck squamous cell carcinoma; LUSC, lung squamous cell carcinoma; ESCCa, esophageal squamous cell carcinoma; 95% CI, 95% confidence interval; KW, Kruskal–Wallis H statistic; H-L, HNSCCa-LUSC; H-E, HNSCCa-ESCCa; L-E, LUSC-ESCCa. Pairwise *p*-values are Bonferroni-adjusted. * Significant (*p* < 0.05).

**Table 2:** HPV+ vs. HPV− Coagulome Analysis.

Outcome	Group	*n*	Mean ± SD	95% CI	U Statistic	*p*-Value
Composite Coagulome	HPV+	72	10.14 ± 1.30	9.85–10.44	21,823	4.28 × 10^−10^ *
	HPV−	415	11.25 ± 1.39	11.12–11.39		
*F3*	HPV+	72	10.23 ± 1.79	9.82–10.64	21,056.5	2.88 × 10^−8^ *
	HPV−	415	11.47 ± 1.71	11.31–11.64		
*SERPINE1*	HPV+	72	10.38 ± 1.53	10.03–10.74	25,255.5	8.16 × 10^−21^ *
	HPV−	415	12.65 ± 1.60	12.50–12.81		
*SERPINB2*	HPV+	72	9.82 ± 2.00	9.36–10.28	14,703	0.83
	HPV−	415	9.63 ± 2.51	9.39–9.87		

All comparisons were performed using two-sided Mann–Whitney U tests. Values are reported as mean log2 RSEM expression ± SD with 95% CI. Abbrev. HPV, human papilloma virus; SD, standard deviation; 95% CI, 95% confidence interval. * Significant (*p* < 0.05).

**Table 3:** Global Differences in Coagulome Gene Expression Across HNSCCa Primary Sites (Kruskal–Wallis Tests).

Outcome	KW H	*p*-Value
Composite Coagulome	57.97	1.6 × 10^−12^ *
*F3*	57.27	2.25 × 10^−12^ *
*SERPINE1*	58.96	9.80 × 10^−13^ *
*SERPINB2*	5.00	0.17

Kruskal–Wallis tests were used to compare expression across primary tumor sites. Expression values are based on raw TCGA/Xena log2 RSEM data. Groups were included if *n* ≥ 10. * Significant (*p* < 0.05).

**Table 4:** Coagulome Gene Expression by Primary Site Among HNSCCa Tumors (log2 RSEM).

Site	*N* (514)	Outcome	Mean ± SD	95% CI
Oral cavity (C00, C02–C06)	242	Composite Coagulome	11.40 ± 1.35	11.24–11.57
		*F3*	11.74 ± 1.57	11.54–11.94
		*SERPINE1*	12.72 ± 1.58	12.53–12.92
		*SERPINB2*	9.75 ± 2.54	9.43–10.07
Larynx (C32)	118	Composite Coagulome	10.82 ± 1.26	10.60–11.05
		*F3*	10.86 ± 1.69	10.55–11.16
		*SERPINE1*	12.20 ± 1.71	11.89–12.51
		*SERPINB2*	9.42 ± 2.22	9.02–9.82
Oropharynx (C01, C09, C10)	77	Composite Coagulome	10.11 ± 1.33	9.81–10.41
		*F3*	10.16 ± 1.64	9.80–10.53
		*SERPINE1*	10.81 ± 1.95	10.38–11.25
		*SERPINB2*	9.35 ± 2.30	8.84–9.87
Other/overlap/hypopharynx (C13, C14)	77	Composite Coagulome	11.29 ± 1.45	10.96–11.61
		*F3*	11.38 ± 1.92	10.95–11.81
		*SERPINE1*	12.60 ± 1.47	12.27–12.92
		*SERPINB2*	9.88 ± 2.48	9.32–10.43

Values represent mean ± SD log2 RSEM expression from TCGA/Xena with 95% CI. Abbrev.: SD, standard deviation; 95% CI, 95% confidence interval.

**Table 5:** T stage, N stage, and Grade Coagulome Analysis.

Comparison	Outcome	Group	*n*	Mean ± SD	95% CI	U Statistic	*p*-Value
T1–2 vs. T3–4	Composite Coagulome	T1–2	183	11.04 ± 1.34	10.85–11.23	23,201.5	0.27
		T3–4	270	11.16 ± 1.42	10.99–11.33		
T1–2 vs. T3–4	*F3*	T1–2	183	11.41 ± 1.74	11.16–11.66	26,320.3	0.24
		T3–4	270	11.24 ± 1.73	11.04–11.45		
T1–2 vs. T3–4	*SERPINE1*	T1–2	183	12.11 ± 1.84	11.84–12.37	20,918.5	0.0056 *
		T3–4	270	12.59 ± 1.57	12.41–12.78		
T1–2 vs. T3–4	*SERPINB2*	T1–2	183	9.60 ± 2.30	9.27–9.93	23,759.5	0.49
		T3–4	270	9.65 ± 2.54	9.35–9.95		
N0 vs. N1–3	Composite Coagulome	N0	174	11.29 ± 1.35	11.08–11.49	31,172.0	0.014 *
		N1–3	316	11.00 ± 1.45	10.84–11.16		
N0 vs. N1–3	*F3*	N0	174	11.46 ± 1.62	11.22–11.70	29,836.5	0.12
		N1–3	316	11.19 ± 1.80	10.99–11.39		
N0 vs. N1–3	*SERPINE1*	N0	174	12.31 ± 1.58	12.07–12.54	26,339.0	0.44
		N1–3	316	12.37 ± 1.84	12.17–12.58		
N0 vs. N1–3	*SERPINB2*	N0	174	10.09 ± 2.34	9.74–10.44	32,409.5	0.0010 *
		N1–3	316	9.43 ± 2.44	9.16–9.70		
G1–2 vs. G3	Composite Coagulome	G1–2	363	11.29 ± 1.35	11.15–11.43	30,196.5	2.2 × 10^−6^ *
		G3	130	10.56 ± 1.45	10.31–10.81		
G1–2 vs. G3	*F3*	G1–2	363	11.40 ± 1.65	11.23–11.57	26,505.0	0.037 *
		G3	130	10.94 ± 2.02	10.59–11.28		
G1–2 vs. G3	*SERPINE1*	G1–2	363	12.47 ± 1.56	12.31–12.63	26,123.0	0.070
		G3	130	11.98 ± 2.11	11.62–12.34		
G1–2 vs. G3	*SERPINB2*	G1–2	363	9.99 ± 2.28	9.76–10.22	30,524	6.7 × 10^−7^ *
		G3	130	8.75 ± 2.52	8.32–9.19		

All comparisons were performed using two-sided Mann–Whitney U tests. Values are reported as mean log2 RSEM expression ± SD with 95% CI. Abbrev. SD, standard deviation; 95% CI, 95% confidence interval. * Significant (*p* < 0.05).

## Data Availability

Quantitative proteomic data for HNSCCa were obtained from the Clinical Proteomic Tumor Analysis Consortium (CPTAC) via the Proteomic Data Commons (PDC). Protein expression data generated using tandem mass tag (TMT)-based mass spectrometry were downloaded from the CPTAC3 Head and Neck Carcinoma proteome dataset (CPTAC3_Head_and_Neck_Carcinoma_Proteome.tmt11.tsv) on 10 February 2026. Study details for CPTAC have previously been described [12].

## Supplementary Materials

The following supporting information can be downloaded at: https://www.mdpi.com/article/10.3390/cancers18071055/s1, Table S1: (a) Global Differences in Coagulome Gene Expression Across HPV-Negative HNSCCa Primary Sites (Kruskal–Wallis Tests). (b) Coagulome Gene Expression by Primary Site Among HPV-Negative HNSCCa Tumors (log2 RSEM); Table S2: Clinical and Demographic Correlates of Coagulome Expression in HNSCCa; Table S3: Benjamini–Hochberg FDR-adjusted *p*-values for HNSCC-specific coagulome analyses.

## Abbreviations

The following abbreviations are used in this manuscript:

VTE	Venous thromboembolism
GBM	Glioblastoma multiforme
PAAD	Pancreatic adenocarcinoma
HNSCCa	Head and neck squamous cell carcinoma
TME	Tumor microenvironment
TF	Tissue factor
OSCCa	Oral cavity squamous cell carcinoma
CAT	Cancer-associated thrombosis
TCGA	The Cancer Genome Atlas
ESCCa	Esophageal squamous cell carcinoma
LUSC	Lung squamous cell carcinoma
HPV	Human papilloma virus
