# Supplementary material for: Tumor-Specific Pro-Thrombotic Gene Expression in Head and Neck Squamous Cell Carcinoma: A Multi-Cohort Transcriptomic Analysis
Source: Cancers (Basel). 2026 Mar 25;18(7):1055. doi: 10.3390/cancers18071055 (PMC13072147; doi:10.3390/cancers18071055)
Supplement: Supplementary file 1 [file cancers-18-01055-s001.zip › cancers-4205598-supplementary.pdf]

Supplementary Materials

Supplemental Table S1a. Global Differences in Coagulome Gene Expression Across HPV-Negative HNSCCa Primary Sites (Kruskal–Wallis Tests).

Outcome	Kruskal-Wallis H Statistic	p-value
Composite Coagulome	21.59	1.28×10^{-4}*
F3	22.10	6.2×10^{-5}*
SERPINE1	10.33	0.016*
SERPINB2	9.85	0.02*

Kruskal–Wallis tests were used to compare expression across primary tumor sites among HPV-negative HNSCCa tumors. Expression values are based on raw TCGA/Xena log2 RSEM data. Groups were included if $n \geq 10$. *Significant at $p < 0.05$.

Supplemental Table S1b. Coagulome Gene Expression by Primary Site Among HPV-Negative HNSCCa Tumors (log2 RSEM).

Site	N (414)*	Outcome	Mean ± SD	95% CI
Oral cavity (C00, C02 – C06)	217	Composite Coagulome	11.46 ± 1.35	11.28 – 11.64
		F3	11.78 ± 1.57	11.57 – 11.99
		SERPINE1	12.81 ± 1.55	12.60 – 13.02
		SERPINB2	9.78 ± 2.57	9.44 – 10.12
Larynx (C32)	108	Composite Coagulome	10.84 ± 1.29	10.60 – 11.08
		F3	10.92 ± 1.73	10.59 – 11.24
		SERPINE1	12.23 ± 1.70	11.91 – 12.55
		SERPINB2	9.37 ± 2.20	8.96 – 9.79
Oropharynx (C01, C09, C10)	23	Composite Coagulome	10.81 ± 1.42	10.23 – 11.39
		F3	10.99 ± 1.52	10.36 – 11.61
		SERPINE1	12.97 ± 1.59	12.32 – 13.62
		SERPINB2	8.48 ± 2.71	7.38 – 9.59
Other/overlap/hypopharynx (C13, C14)	66	Composite Coagulome	11.39 ± 1.51	11.02 – 11.75
		F3	11.52 ± 1.93	11.06 – 11.99
		SERPINE1	12.71 ± 1.49	12.35 – 13.06
		SERPINB2	9.94 ± 2.58	9.31 – 10.56

Values represent mean ± SD log2 RSEM expression from TCGA/Xena with 95% CI among HPV-negative HNSCCa tumors. . *Analytic sample size limited to 414; one tumor lacking sufficient site annotation or gene expression data was excluded in the final analysis. . Abbrev. KW, Kruskal-Wallis H statistic; SD, standard deviation; 95% CI, 95% confidence interval.

Supplemental Table S2. Clinical and Demographic Correlates of Coagulome Expression in HNSCCa.

Variable	Comparison	Outcome	Test	Test Statistic	p-value
**Age**	Continuous	Composite Coagulome	Spearman correlation	$\rho < 0.10$	>0.10
		F3	Spearman correlation	$\rho < 0.10$	>0.10
		SERPINE1	Spearman correlation	$\rho < 0.10$	>0.10
		SERPINB2	Spearman correlation	$\rho < 0.10$	>0.10

**Sex**	Male (n = 350) vs Female (n = 180)	Composite Coagulome	Mann-Whitney U	U = 15,002	0.28
		F3	Mann-Whitney U	U = —	>0.30
		SERPINE1	Mann-Whitney U	U = —	>0.30
		SERPINB2	Mann-Whitney U	U = —	>0.30
**Race**	White (n = 452) vs Non-White (n = 63)	Composite Coagulome	Kruskal-Wallis	H < 5.0	>0.10
		F3	Kruskal-Wallis	H < 5.0	>0.10
		SERPINE1	Kruskal-Wallis	H < 5.0	>0.10
		SERPINB2	Kruskal-Wallis	H < 5.0	>0.10

Values were analyzed using log2 RSEM expression values from TCGA/Xena. Age correlations were assessed using Spearman rank correlation (ϱ). Sex comparisons were performed using two-sided Mann–Whitney U tests, and race comparisons using Kruskal–Wallis tests.

Supplemental Table S3. Benjamini–Hochberg FDR-adjusted p-values for HNSCC-specific coagulome analyses.

**Analysis**	**Outcome**	**p-value**	**FDR-adjusted p-value**	**Significant after FDR**
HPV status	Composite Coagulome	4.28×10^{-10}	2.14×10^{-9}*	Yes
HPV status	*F3*	2.88×10^{-8}	9.60×10^{-8}*	Yes
HPV status	*SERPINE1*	8.16×10^{-21}	1.63×10^{-19}*	Yes
HPV status	*SERPINB2*	0.83	0.83	No
Primary site	Composite Coagulome	1.6×10^{-12}	1.07×10^{-11}*	Yes
Primary site	*F3*	2.25×10^{-12}	1.13×10^{-11}*	Yes
Primary site	*SERPINE1*	9.8×10^{-13}	8.17×10^{-12}*	Yes
Primary site	*SERPINB2*	0.17	0.23	No
T stage	Composite Coagulome	0.27	0.32	No
T stage	*F3*	0.24	0.32	No
T stage	*SERPINE1*	0.0056	0.016*	Yes
T stage	*SERPINB2*	0.49	0.52	No
N stage	Composite Coagulome	0.014	0.031*	Yes
N stage	*F3*	0.12	0.17	No
N stage	*SERPINE1*	0.44	0.49	No
N stage	*SERPINB2*	0.0010	0.0040*	Yes
Grade	Composite Coagulome	2.2×10^{-6}	7.3×10^{-6}*	Yes
Grade	*F3*	0.037	0.068	No
Grade	*SERPINE1*	0.070	0.11	No
Grade	*SERPINB2*	6.7×10^{-7}	2.7×10^{-6}*	Yes
Primary site (HPV–)	Composite Coagulome	1.28×10^{-4}	3.2×10^{-4}*	Yes
Primary site (HPV–)	*F3*	6.2×10^{-5}	2.1×10^{-4}*	Yes
Primary site (HPV–)	*SERPINE1*	0.016	0.032*	Yes
Primary site (HPV–)	*SERPINB2*	0.020	0.036*	Yes

Benjamini–Hochberg FDR correction was applied across all HNSCC-specific hypothesis tests reported in Tables 2–4 and Supplemental Table S1a. Abbrev. HPV, human papilloma virus; T, tumor; N; nodal; FDR, false discovery rate. *FDR-adjusted p < 0.05.
